# Hydroxyurea promotes TET1 expression and induces apoptosis in osteosarcoma cells

**DOI:** 10.1042/BSR20190456

**Published:** 2019-05-14

**Authors:** Songsong Teng, Chunhui Ma, Yinxian Yu, Chengqing Yi

**Affiliations:** Department of Orthopedics, Shanghai General Hospital, Shanghai Jiao Tong University School of Medicine, Shanghai, China

**Keywords:** cancer, cell apoptosis, cell cycle, hydroxyurea, TET1

## Abstract

Ten-eleven translocation (TET) proteins are abnormally expressed in various cancers. Osteosarcoma cells were treated with hydroxyurea to investigate the expression pattern of TET proteins in these cells. The expression of TET1 was increased in U2OS cells after treatment with hydroxyurea. In addition, hydroxyurea increased cell apoptosis and altered the cell cycle. TET proteins catalyze the oxidation of 5-methylcytosine (5mC) to 5-hydroxymethylcytosine (5hmC); therefore, 5mC and 5hmC levels were evaluated. Increased 5hmC levels were observed after the hydroxyurea treatment. Experiments examining cell apoptosis and the cell cycle after knockdown and overexpression of TET1 were conducted to further investigate whether TET1 expression affected cell growth. The overexpression of TET1 increased cell apoptosis and inhibited cell growth. Taken together, TET1 expression regulated proliferation and apoptosis in U2OS cells, changes that were associated with 5hmC levels.

## Introduction

Osteosarcoma (OS) is the most common type of bone cancer in both adolescents and young adults [1]. Abnormal epigenetic modifications, such as DNA methylation, have been observed in OS cells [2,3]. Ten-eleven translocation (TET) proteins, which catalyze the oxidation of 5-methylcytosine (5mC) to 5-hydroxymethylcytosine (5hmC), play a crucial role in global epigenetic modifications [4]. Accumulating evidence has revealed TET1-mediated DNA demethylation in hepatocarcinoma cells [5]. Moreover, TET2 inhibits tumorigenesis by up-regulating caspase-4 expression in breast cancer cells [6]. In a recent study, TET3 was proposed to function as an hsa-miR-150 target and to play a role in chronic myelomonocytic leukemia (CML) [7]. Based on these results, TET proteins are closely associated with cancer development.

Hydroxyurea (HU) is the recommended anti-cancer medicine that affects the cell cycle by inhibiting DNA synthesis in a reversible manner [8]. HU has been suggested as the first line therapy for CML [9]. Moreover, in a variety of studies, HU has been utilized to treat bladder cancer, breast cancer, melanoma, gastric cancer, colorectal cancer and hepatocellular carcinoma [10,11]. HU inhibited cancer development in these studies.

In the present study, we aimed to evaluate TET gene expression profiles, including TET1, TET2, and TET3, in U2OS cells following treatment with HU. In addition, cell apoptosis, cell cycle, and cell growth were analyzed in cells with knockdown and overexpression of TET1.

## Materials and methods

### Cell culture and drug treatment

The hFoB and U2OS OS cell lines were cultured in high glucose Dulbecco’s Modified Eagle’s Medium (DMEM) (Gibco, CA, U.S.A.) supplemented with 10% fetal bovine serum (FBS, Gibco, CA, U.S.A.) at 37°C in a 5% CO_2_ atmosphere. Cells (2 × 10^5^ cells/ml) were seeded in six-well plates, after which HU was added and incubated with cells for 48 h.

### Knockdown and overexpression of *TET1*

Synthetic RNA oligonucleotides targeting *TET1* were obtained from RiboBio (Guangzhou, China). The siRNA sequence targeting *TET1* was GCACGCATGAATTTGGATA. FH-TET1-pEF was purchased from Addgene (Addgene plasmid # 49792, MA, U.S.A.). Cells were transfected with si-TET1 and FH-TET1-pEF using liposomes (Lipofectamine 3000, Invitrogen, CA, U.S.A.) for 48 h. As a control, cells were transfected with a non-specific, scrambled siRNA.

### Gene expression analysis

Total RNA was extracted from U2OS cells using the TRNzol reagent (TIANGEN, Beijing, China) according to the manufacturer’s instructions. Extracted RNA was first treated with DNase I (Fermentas, Ontario, Canada) and then reverse transcribed into cDNAs using the BioRT cDNA first strand synthesis kit (Bioer Technology, Hangzhou, China). Quantitative real-time PCR (qPCR) was performed to measure gene expression. Primer sequences used in this study are listed in Supplementary Table S1. qPCR was performed using the BIO-RAD iQ5 Multicolor Real-Time PCR Detection System with the BioEasy SYBR Green I Real Time PCR Kit (Bioer Technology, Hangzhou, China). The following PCR conditions were used: 95°C for 3 min, followed by 40 cycles of denaturation at 95°C for 10 s, annealing at 60°C for 15 s, and extension at 72°C for 30 s. The 2^−ΔΔCT^ method was used to determine relative gene expression, which was normalized to the expression of the *GAPDH* mRNA. For each gene, experiments were performed in triplicate. Data are presented as means ± S.E.M.

### Western blot analysis

For Western blot analysis, proteins were extracted from cells using 2× SDS lysis buffer. Protein concentrations were determined using the BCA protein assay kit (TIANGEN, Beijing, China). Proteins were separated on a 10% SDS-polyacrylamide gel and transferred to a PVDF membrane. Then, membranes were blocked with 5% nonfat milk powder in TBS-T (0.1% Tween-20 in PBS) and incubated with primary antibodies overnight at 4°C. The primary antibodies used in the present study were rabbit anti-TET1, anti-TET2, anti-TET3, and mouse anti-GAPDH antibodies (Abcam, Cambridge, U.K.). After washes with PBS-T, membranes were incubated with horseradish peroxidase (HRP)-conjugated secondary antibodies (anti-mouse or anti-rabbit, Invitrogen, Waltham, U.S.A.) for 1 h at room temperature. Then, proteins were visualized using ECL Super Signal reagent (Pierce, Rockford, U.S.A.).

### Cell counting kit-8 assay

A Cell Counting Kit-8 (CCK8) assay kit (Dojindo, Kumamoto, Japan) was used as previously described [12]. Briefly, cells were plated in 96-well plates at a density of 4 × 10^3^ cells/well. Then, cells were cultured for 48 h, and 10 μl of CCK8 solution was added to the cells and incubated for 2.5 h at 37°C. The absorbance was measured at 450 nm using a microplate reader (Infinite M200, TECAN, Switzerland).

### Cell cycle and apoptosis analyses

For the analysis of the cell cycle, propidium iodide (PI) staining was performed. Briefly, U2OS cells (1 × 10^6^ cells/ml) were treated with HU, siRNA, or FH-TET1-pEF for 48 h. Then, cells were washed with PBS and fixed with 70% ethanol for 2 h at 4°C. Cells were incubated with PI and RNase A for 30 min, and an Accuri™ C6 flow cytometer was used to analyze the cell cycle.

The procedure for detecting apoptotic cells was described previously [13]. Briefly, U2OS cells were subjected to Annexin V-FITC/PI staining after treatment with HU for 48 h. Following the incubation, cells were washed twice with PBS, harvested, and resuspended at a density of 1 × 10^6^ cells/ml Annexin V-FITC, and PI staining was performed according to the manufacturer’s instructions. Cells were incubated for 30 min and subsequently analyzed using an Accuri™ C6 flow cytometer (BD Biosciences, Franklin Lakes, NJ, U.S.A.).

### Immunofluorescence staining

For immunofluorescence staining, cells were plated on coverslips and fixed with a 4% paraformaldehyde solution for 15 min at room temperature. After fixation, cells were washed with PBS and permeabilized with PBS containing 0.5% Triton X-100 for 30 min. Cells were incubated with 4 M HCl for 15 min to denature the DNA, rinsed with distilled water, and placed in 100 mM Tris-HCl (pH 8.5) for 10 min. After washes with PBS, non-specific binding sites were blocked with blocking buffer (10% FCS and 0.1% Tween-20 in PBS) for 1 h. Next, cells were incubated with 5mC (1:100, Cell Signaling Technology, Danvers, U.S.A.) or 5hmC (1:100, Cell Signaling Technology, Danvers, U.S.A.) overnight at 4°C. Subsequently, cells were washed with PBS three times for 10 min each, followed by an incubation with Alexa Fluor 488- or 594-conjugated secondary (anti-mouse or anti-rabbit) antibodies for 1 h at room temperature. Cells were washed three times with PBS for 10 min each, and the coverslip was mounted on a glass slide using an anti-fade mounting medium (BOSTER, China). Cells were imaged using a fluorescence microscope. Image analysis software (ImageJ) was used to evaluate the average fluorescence intensity (AFI) in the cells.

### Statistical analysis

The qPCR and flow cytometry (FCM) data were analyzed with *t* tests using SPSS 16.0 software (SPSS Inc., Chicago, IL, U.S.A.). *P*<0.05 was considered statistically significant.

## Results

### Analysis of TET expression after HU treatment

**Figure 1:**
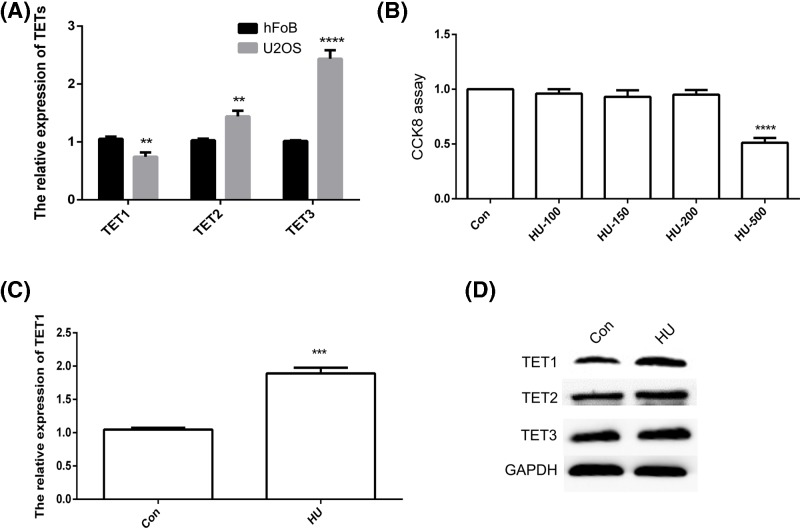
The expression patterns of *TET1, TET2*, and *TET3* The relative expression of *TET1, TET2*, and *TET3* was analyzed using qPCR (**A**). Cell growth was analyzed using the CCK8 assay (**B**). The relative expression of *TET1* in hydroxyurea (HU)-treated U2OS cells was analyzed using qPCR (**C**). TET1, TET2, and TET3 levels in U2OS cells were analyzed using Western blotting (**D**). Con indicates the control. The data are presented as means ± S.E.M. (*n*=3). **P<0.01, ***P<0.005, and ****P<0.001 indicate statistically significant differences.

**Figure 2:**
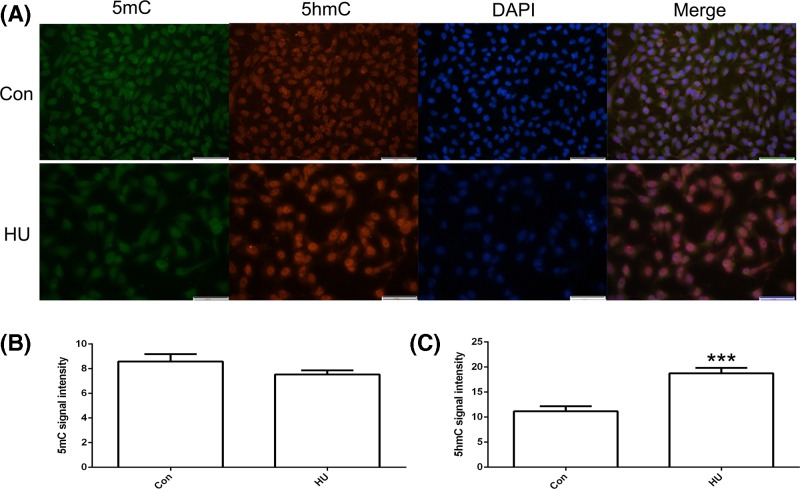
Immunofluorescence localization of 5mC and 5hmC after the hydroxyurea (HU) treatment The expression of 5mC and 5hmC in U2OS cells (**A**). 5mC (**B**) and 5hmC (**C**) signal intensities were measured. Green indicates 5mC. Red indicates 5hmC. Blue indicates DAPI. Con indicates the control. The bar represents 50 μm. ***P<0.005 indicates statistically significant difference.

The expression of *TET1, TET2*, and *TET3* was studied in hFoB and U2OS cells using qPCR. *TET1* expression was reduced and *TET2* and *TET3* expression were increased in U2OS cells (Figure 1A). A CCK8 assay was performed to determine whether HU affected the growth of U2OS cells. The treatment with 500 μM HU exerted a toxic effect on U2OS cells. Therefore, 200 μM HU was used for qPCR and Western blot analyses (Figure 1B). The qPCR results indicated increased *TET1* expression in U2OS cells after the HU treatment (Figure 1C). The results of the Western blot analysis were consistent with the qPCR data (Figure 1D). We analyzed 5mC and 5hmC levels to further confirm the TET1 protein expression pattern. As expected, increased 5hmC levels were observed after the HU treatment (Figure 2A). The statistical analysis confirmed the AFI data (Figure 2B,C). Based on these results, TET1 expression in U2OS cells was regulated by HU, which increased 5hmC levels.

### HU induced cell apoptosis and increased 5hmC levels

**Figure 3:**
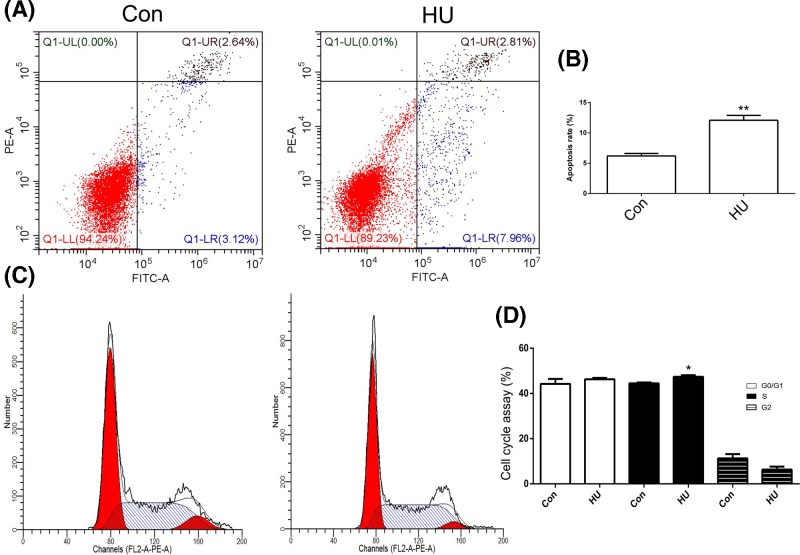
Analysis of cell apoptosis and cell cycle phases Cell apoptosis was analyzed after treatment with hydroxyurea (HU) (**A**). Statistical analysis of the percentage of apoptotic cells (**B**). The cell cycle phases were analyzed after the HU treatment (**C**). Statistical analysis of the percentage of cells in each phase of the cell cycle (**D**). Con indicates the control. *P<0.05 and **P<0.01 indicate statistically significant differences.

Compared with the control, the HU treatment significantly increased the percentage of apoptotic U2OS cells (Figure 3A,B). In addition, the effects of the HU treatment on the cell cycle profile were analyzed. A significantly increased number of HU-treated cells were observed in S phase (Figure 3C,D). Thus, the HU treatment altered the cell cycle and cell apoptosis profiles of U2OS cells.

### *TET1* expression regulated cell apoptosis

**Figure 4:**
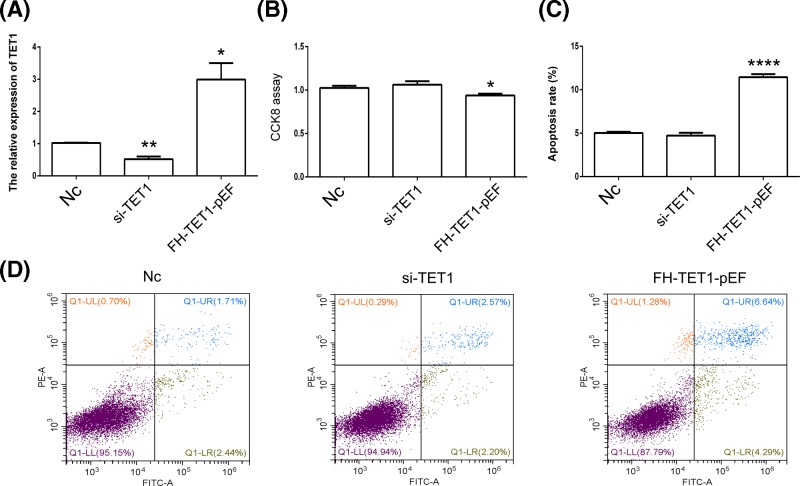
Analysis of cell growth and cell apoptosis *TET1* expression in U2OS cells was analyzed using qPCR (**A**). Cell growth was analyzed using the CCK8 assay (**B**). Analysis of cell apoptosis after the hydroxyurea treatment (**C** and**D**). Nc indicates the negative control. *P<0.05, **P<0.01 and ****P<0.001 indicate statistically significant differences.

**Figure 5:**
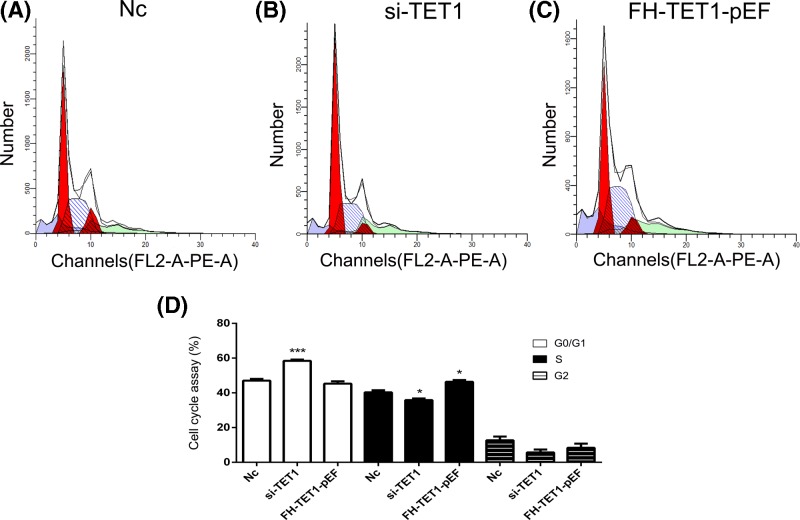
Analysis of the cell cycle Analysis of the cell cycle in the Nc (**A**), si-TET1 (**B**), and FH-TET1-pEF (**C**) groups. Statistical analysis of the percentage of cells in each phase of the cell cycle (**D**). Nc indicates the negative control. *P<0.05 and ***P<0.005 indicate statistically significant differences.

**Figure 6:**
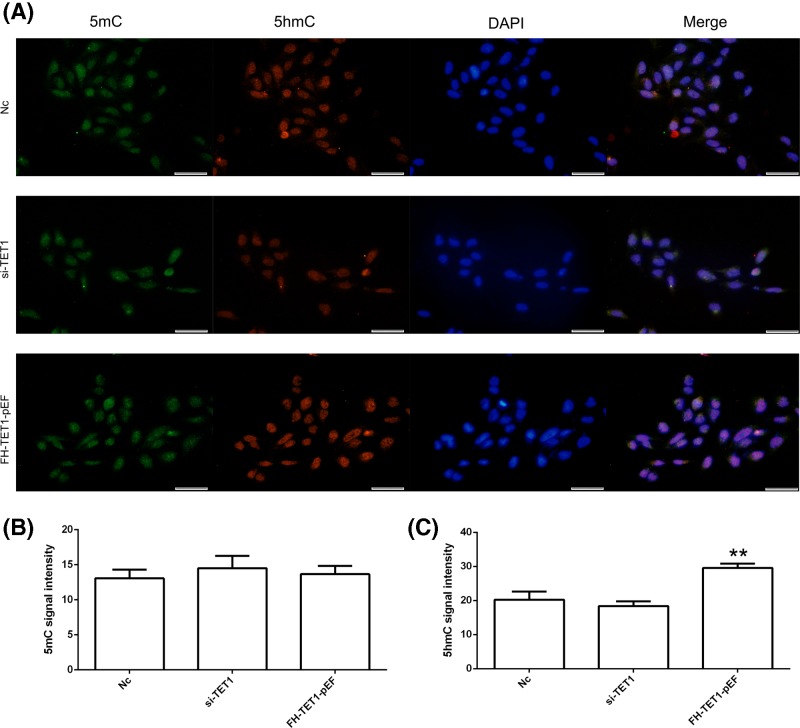
Immunofluorescence localization of 5mC and 5hmC The expression of 5mC and 5hmC in U2OS cells (**A**). 5mC (**B**) and 5hmC (**C**) signal intensities were measured. Green indicates 5mC. Red indicates 5hmC. Blue indicates DAPI. Nc indicates the negative control. The bar represents 50 μm. **P<0.01 indicates statistically significant difference.

Knockdown and overexpression of *TET1* were performed to further analyze the effects of *TET1* expression. The qPCR results showed a reduction in *TET1* expression in the si-TET1 group, while *TET1* overexpression was observed in the FH-TET1-pEF group (Figure 4A). According to the results of the CCK8 assay, *TET1* overexpression inhibited cell growth (Figure 4B). The results of the cell apoptosis assay showed that *TET1* overexpression induced cell death (Figure 4C,D). In addition, *TET1* expression altered the cell cycle in U2OS cells (Figure 5). Moreover, the global DNA methylation level indicated increased levels of 5hmC following the overexpression of TET1 (Figure 6A). The statistical analysis confirmed our AFI results (Figure 6B,C). Thus, *TET1* expression played a role in regulating cell apoptosis and the cell cycle.

## Discussion

In previous studies, TET proteins were reported to play an important role in various cancer cells, including HepG2, A549, and MCF7 cells [14–16]. In the present study, we analyzed the expression of *TET1, TET2*, and *TET3* in hFoB and U2OS cells, and our results revealed abnormal expression of *TET1* in U2OS cells. Due to the important role of the TET1 protein in DNA methylation, this finding might indicate that U2OS cells exhibit an aberrant methylation status. Indeed, abnormal DNA methylation has been observed in numerous cancer cells [17,18]. Moreover, in various studies, abnormal DNA methylation is closely associated with TET protein expression [19,20]. Based on these results, abnormal TET1 expression was confirmed in OS cells and therefore may affect DNA methylation. In addition, U2OS cells were treated with HU to analyze the *TET1* expression pattern. In previous studies, HU has been used to treat myeloproliferative diseases, sickle cell anemia [21], melanoma and ovarian carcinoma [22]. HU regulated *TET1* expression in the present study, suggesting that it might have a role in osteosarcoma. In the present study, abnormal *TET1* expression was confirmed in OS cells and affected DNA methylation.

In previous report, abnormal global DNA methylation, which was regulated by TET proteins, was observed in cancer cells [23]. Compared with 5mC, 5hmC activates gene expression and is mainly enriched in gene bodies. In recent studies, a loss of 5hmC has been observed in many cancers [24,25]. Thus, overexpression and knockdown of *TET1* expression were used to evaluate 5hmC levels in U2OS cells. *TET1* overexpression increased 5hmC levels, which were decreased by si-TET1. Thus, 5hmC played an important role in the biological processes of U2OS cells.

*TET1* is involved in the growth, apoptosis, and cell cycle of cancer cells [20]. HU, an anti-tumor drug, has been shown to inhibit cell growth, induce cell apoptosis, and alter the cell cycle [9]. In the present study, the HU treatment increased *TET1* expression and induced apoptosis in U2OS cells. Therefore, cell growth, cell apoptosis, and the cell cycle were analyzed following knockdown and overexpression of *TET1. TET3* regulates the proliferation of HepG2 cells [26]. Compared with hepatocellular carcinoma, our results indicated that increased *TET1* expression induced apoptosis and inhibited the growth of U2OS cells, consistent with previous data obtained from colon cancer cells [27]. *TET1* expression was recently reported to be associated with apoptosis in bladder cancer cells [28]. As shown in the present study, *TET1* overexpression induced apoptosis in HU-treated cells. Moreover, the TET1 protein has been suggested that play a role in the cell cycle [29]. Our result confirmed that changes in *TET1* expression altered the cell cycle in U2OS cells. Together, TET1 regulated 5hmC levels and may play a crucial role in the biological processes of cancer cells.

## Conclusions

In conclusion, TET1 expression in U2OS cells is regulated by HU. Overexpression of *TET1* increased 5hmC levels, induced cell apoptosis, and altered the cell cycle. Taken together, our data indicate an important role for TET1 in cancer development and that TET1 represents a potentially novel biomarker in cancer therapy.

## Supporting information

**Supplemental Table S1:** Primers for si-RNA analysis

**Supplemental Table S2:** Primers for qRT-PCR analysis

## Abbreviations

5hmC: 5-hydroxymethylcytosine
5mC: 5-methylcytosine
OS: osteosarcoma
TET: ten-eleven translocation
