# Supplementary material for: Hydroxyurea promotes TET1 expression and induces apoptosis in osteosarcoma cells
Source: Biosci Rep. 2019 May 14;39(5):BSR20190456. doi: 10.1042/BSR20190456 (PMC6522705; doi:10.1042/BSR20190456)
Supplement: Supplementary file 1 [file BSR-2019-0456_suppS1.pdf]

Table

Table S1 Primers for si-RNA analysis

siRNA	Target sequence
si-TET1	GCACGCATGAATTTGGATA

Table S2 Primers for qRT-PCR analysis

Genes	Annealing (°C)	Primer sequences (5’→3’)	Reference/accession
TET1	60	F: CAGAACCTAAACCACCCGTG<br>R: TGCTTCGTAGCGCCATTGTAA	(Yang et al., 2015)
TET2	60	F: GATAGAACCAACCATGTTGAGGG<br>R: TGGAGCTTTGTAGCCAGAGGT	(Yang et al., 2015)
TET3	60	F: TCCAGCAACTCCTAGAACTGAG<br>R: AGGCCGCTTGAATACTGACTG	(Yang et al., 2015)
GAPDH	60	F: TGGTATCGTGGAAGGACTCA<br>R: GGGCCATCGACAGTCTTC	J02642.1
